# Virtual City for Exposure Therapy in Phobias: Case Studies of Agoraphobia

**DOI:** 10.1192/j.eurpsy.2023.460

**Published:** 2023-07-19

**Authors:** M. Jablonská, A. Francová, K. Janků, P. Stopková, E. Nosková, I. Fajnerová

**Affiliations:** ^1^ Research Center for Virtual Reality in Mental Health and Neuroscience; ^2^Inpatient Ward for Anxiety Disorders, National Institute of Mental Health in Czech Republic, Prague, Czech Republic

## Abstract

**Introduction:**

A phobia is a type of anxiety disorder that causes an individual to experience extreme, irrational fear about a situation, living creature, place, or object. The most common in the treatment of phobic disorders are in vivo exposures (IVEs) consisting of confrontation with feared stimulus until distress has decreased. Virtual reality exposure therapy (VRET) is a modern alternative to IVEs where patients are exposed to virtual anxiety-provoking environments, and its effectiveness has already been demonstrated in the treatment of most phobias (Freitas et al. Psychiatr q. 2021; 92(4):1685–1710).

**Objectives:**

This paper aims to present a complex virtual city developed for VRET in different types of phobia. The VRET system is composed of several interactive environments (a skyscraper, a subway, a cinema, and a hospital) that can be combined in form of different scenarios targeting various phobias, allowing controlled and gradual exposure. Selected virtual environments will be presented in case studies of agoraphobic patients (F40 by ICD-10).

**Methods:**

The number of VRET sessions is individual, based on the need of each patient, starting with an introductory session including stimulus mapping and VR control explanation. Each session lasts about 30 minutes. During exposure, Subjective Units of Discomfort (SUDS) are assessed at various points. The scenarios for agoraphobia are typically composed of an elevator or subway ride, open spaces (city streets, the roof ), or crowded interiors (the cinema). Environments allow various effects, elevator trembling, getting stuck situations and adjusting the number of people. All the scenes contain authentic ambient sounds.

**Results:**

We present a case of a 33-year-old male patient experiencing intense fear of getting stuck or locked, turning into panic attacks. During 10 VRET sessions, the patient was exposed to different environments (subway, underground parking, elevator, cinema) focusing primarily on elevator rides, sometimes in combination with IVE, consisting of locking the door in the experimental room. Another case is a 59-year-old female patient with a fear of open spaces and crowd situations. This patient had 5 VRET sessions combining exposure to open spaces with subway rides. In addition, the patient was instructed to watch short 360° videos of crowd situations. The last case is a 20-year-old female patient with an intense fear of subway tunnels and sounds, enclosed spaces, and heights. During 4 VRET sessions, we mainly focused on the subway with additional IVEs in a real elevator.

**Conclusions:**

Subjective evaluations during exposures indicate a reduction of anxiety across sessions. Based on the patient’s feedback we can conclude on a good acceptance of the technology and an improvement in real-life situations. These case studies demonstrate the valuable use of variable combinations of virtual scenarios in the treatment of agoraphobia.

**Disclosure of Interest:**

None Declared

